# Sir Humphry Davy and the coal miners of the world: a commentary on Davy (1816) ‘An account of an invention for giving light in explosive mixtures of fire-damp in coal mines’

**DOI:** 10.1098/rsta.2014.0288

**Published:** 2015-04-13

**Authors:** John Meurig Thomas

**Affiliations:** Department of Materials Science and Metallurgy, University of Cambridge, 27 Charles Babbage Road, Cambridge CB3 0FS, UK

**Keywords:** fire-damp, coal mines, safety lamp, science of combustion

## Abstract

In the period between 1815 and 1818, Sir Humphry Davy read four papers to the Royal Society and published a monograph dealing with a safety lamp for coal miners, all of which record in detail the experimental work that he carried out, with his assistant Michael Faraday, so as to determine how to prevent catastrophic accidents in coal mines by the explosion of fire-damp (methane) in the presence of a naked flame. This article describes the key experiments that he performed at the Royal Institution and some of the subsequent trials made in the coal mines of the north of England. It begins, however, with an account of Davy's prior achievements in science before he was approached for help by the clergymen and doctors in the Gateshead and Newcastle upon Tyne areas. There is little doubt that the Davy lamp, from the 1820s onwards, transformed the coal industry worldwide. It also profoundly influenced the science of combustion, and in the words of a pioneer in that field, W. A. Bone, FRS, ‘There is no better model of logical experimental procedure, accurate reasoning, philosophical outlook and fine literary expression.’ It is a remarkable fact that it took Davy essentially only two weeks from the time he was given samples of fire-damp to solve the problem and to devise his renowned miner's safety lamp. A brief account is also given of the contemporaneous invention of a safety lamp by George Stephenson, and of some of Davy's subsequent accomplishments. This commentary was written to celebrate the 350th anniversary of the journal *Philosophical Transactions of the Royal Society*.

## 1. Sir Humphry Davy (1778–1829)

So much has been written about the life and work of Humphry Davy that it is hardly necessary to expatiate further on his extraordinary range of accomplishments. Detailed memoirs by Paris [1] and his brother, John Davy [2], are well known—and so are some of their faults. An admirable biography, by T. E. Thorpe, FRS, appeared in 1896 [3], and a concise and illuminating account of all aspects of his life and work is given in the monograph by Sir Harald Hartley, FRS [4]. Equally useful accounts of Davy's life and work are also available [5–7]. Richard Holmes' admirable *The age of wonder* [8] details many of Davy's interests and activities, and recent short accounts of Davy as a natural philosopher, discoverer, inventor, poet and man of action have been published by this author and his colleagues [9,10].

Davy was, arguably, the first-ever popularizer of chemical science: he combined felicity of literary and poetic expression with brilliant scientific discovery and demonstration. The intellectuals, socialites and aristocrats of London flocked in droves in the early decades of the nineteenth century to his lectures at the Royal Institution (RI). Such was the congestion caused by the numerous carriages that entered Albemarle Street in the heart of Mayfair (less than a mile from Piccadilly Circus) that it became the first one-way street in the metropolis. Davy's pellucid presentations on science were the talk of the town. In one of his lecture-demonstrations, on an introduction to ‘electro chemical science’, given on 12 March 1808, he began with the following sentences:

It affords me the most sincere pleasure to be able to appear again before you. There is no desire more alive and ardent in my mind than that of having it in my power to combine experiments made for the advancement of science, with the details of public lectures…

Not only were his lectures and demonstrations vividly presented, his range of scientific accomplishments was extraordinary. By the time a London barrister, Mr J. J. Wilkinson, representing individuals such as Reverend Dr John Hodgson, vicar in the district where, in 1812, 92 men and boys were killed in a colliery explosion, and the Bishop of Durham, appealed for his assistance in 1815, Davy had already made the following discoveries and scientific advances:

(i) Opened up the field of electrochemistry. He had read Volta's famous memoir *On the generation of continuous electricity by the mere contact of two dissimilar metals* (addressed to Sir Joseph Banks, PRS, in 1800) and concluded that it was not physical contact between dissimilar metals that was the source of electricity; rather, he believed it was chemical action. In 1800, Davy published five papers in which he demonstrated conclusively that chemical action is indeed the root cause of the generated electricity. These experiments won him great acclaim from the eminent Swedish chemist Berzelius. And when Davy ‘inverted’ the argument, i.e. argued that electricity could cause chemical reactions, it led him to the discovery and isolation of alkali and alkaline earth metals. In due course, he:
(ii) discovered sodium, potassium, calcium, barium, strontium, magnesium and boron;
(iii) demonstrated that chlorine was an element;
(iv) discovered the anaesthetic properties of nitrous oxide (laughing gas);
(v) determined the capacity of the human lung; he was also the first to record the medical condition now known as laryngospasm, which he discovered in endeavouring to breathe pure carbon dioxide;
(vi) invented the carbon arc, used for street lighting;
(vii) demolished Lavoisier's contention that all acids contain oxygen; the so-called oxymuriatic acid turned out to be HCl, hydrogen chloride. And he had also:
(viii) laid the foundation of agricultural chemistry; he had worked out the productivity and nutritional value of various grasses and also the nutrients required to be added to soil to facilitate growth; his book *Elements of Agricultural Chemistry*, published in 1813, contained 97 appendices detailing the yields and other qualities of various grasses;
(ix) written, in 1803, an illuminating account of the constituent parts of certain astringent vegetables, and their operation in the tanning of animal skins; this account, as well as a few others, earned him the Society's premier award, the Copley Medal, in 1805.

## 2. Davy's approach to the invention of the miner's safety lamp

Davy was indulging in his sporting activities in the Highlands of Scotland (throughout his life, from his early teens, he frequently took time off to go fishing and hunting) when the plea for help came from Mr Wilkinson and Dr Gray (rector of Bishopwearmouth—later Bishop of Bristol). He replied immediately and said he would visit Newcastle on his return journey so as to investigate the problem. This he did in August 1815, when he spent some days in discussion with colliery owners and, in particular, with Mr Buddle (of the Wallsend Colliery)—an influential person on Tyneside and County Durham—who had endeavoured to minimize the risk of explosions by forced ventilation of his mines.

Davy quickly ascertained the nature of the problem, and he acquired, for his return to the RI, bulk samples of fire-damp from various sources in the mines. In his laboratory, he reassured himself that fire-damp was, indeed (as had been demonstrated earlier by Henry and by Dalton), methane, CH_4_. He set about investigating the range of concentrations in which fire-damp formed an explosive mixture with air, and then focused his attention on the degree of heat needed to ignite it. He discovered that fire-damp was relatively harder to ignite than explosive mixtures of air with H_2_ or carbonic oxide (CO) or olefiant gas (ethylene, C_2_H_4_). For the ignition of fire-damp, a higher temperature was required. Next, he studied the expansion that occurred on the explosion of various mixtures and their power of communicating flame through apertures to other explosive mixtures. These experiments yielded the clue that led him to the ultimate solution. He investigated the movement of the flame of an explosive mixture of coal gas (which consists mainly of H_2_, CO and C_2_H_4_) and air in a tube one-quarter of an inch in diameter and one foot long. He found that it took a second to travel from one end to the other. When the diameter of the tube was reduced to one-seventh of an inch he found that he could not make the mixture explode, although coal gas was more explosive than fire-damp. He then exploded mixtures of fire-damp via a jar connected with a bladder filled with the same mixture by means of a stopcock with an aperture of one-sixth of an inch and found that the flame did not ignite the gas in the bladder. On comparing the effect of connections between the jar and the bladder made of glass and metal, he noted that flames passed more readily through glass tubes than metal tubes of the same diameter, a fact that he attributed to the higher thermal conductivity of the metal and, hence, the cooling it produced, bearing in mind his earlier observation that—in his own words—*the fire-damp requires a very strong heat for inflammation*.

Davy also established that the explosion would not pass through metal slots if their diameter was less than one-seventh of an inch, provided they were of sufficient length, nor would it pass through fine wire-gauze. The latter was an important discovery. He then examined the effect of mixing CO_2_ or N_2_ (azote) with the explosive mixture and found that the presence of one part of N_2_ in six parts of the explosive mixture deprived it of its explosive power. He obtained the same result with one part of CO_2_ in seven parts of the mixture. He concluded that this effect was attributable to the cooling of the flame by this admixture of an ‘inert’ gas.

Equipped with all of these fundamental, experimental facts, Davy possessed sufficient evidence to design his first safety lamp in which, by admitting only a limited supply of air to an oil burner, in a closed lantern, the amount of CO_2_ and N_2_ would be sufficient to prevent an explosion if the air were contaminated with fire-damp. Then came his crucial test. With the lamp described above, he tested it with the lantern supplied by air through two glass tubes one-tenth of an inch in diameter, the chimney being protected in a similar manner. He then introduced this lighted lantern into a large jar containing an explosive mixture of air and fire-damp: the flame simply increased in size and then was extinguished without causing the mixture in the jar to explode.

It is instructive to quote what Davy wrote in his first paper [11, pp. 11–12] that he read to the Royal Society on 9 November 1815—*just two weeks or so* after addressing and solving the major problem that he agreed to investigate:

It is evident, then, that to prevent explosions in coal mines, it is only necessary to use air-tight lanterns, supplied with air from tubes or canals of small diameter, or from apertures covered with wire gauze placed below the flame, through which explosives cannot be communicated, and having a chimney at the upper part, on a similar system for carrying off the foul air; and common lanterns may be easily adapted to the purpose, by being made air-tight in the door and sides, by being furnished with the chimney, and the system of safety apertures below and above.

**Figure 1.**
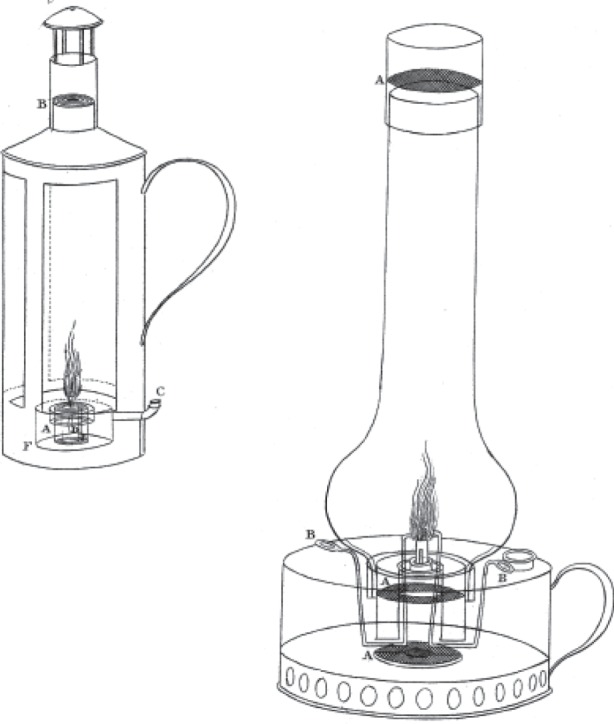
Images are figs. 1 (left) and 9 (right) of Davy's paper [11]. The left hand image represents the safe lantern with its air-feeder and chimney furnished with safety metallic canals. It contains about a quart of air. The sides are of horn or glass, made air tight by putty or cement. (A) is the lamp through which the circular air-feeding canals pass: they are 3 concentric hollow cylinders, distant from each other 1/26 of an inch: the smallest is 2 1/2 inches in circumference; their depth is 2 inches. (B) is the chimney containing 4 such canals, the smallest 2 inches in circumference: above it is a hollow cylinder, with a cap to prevent dust from passing into the chimney. (C) is the hole for admitting oil. (D) is a long canal containing a wire by which the wick is moved or trimmed. (E) is the tube forming a connection between the reservoir of oil and the chamber that supplies the wick with oil. (F) is the rim round the bottom of the lantern to enable it to bear motion. Davy's fig. 9 (right) is a metallic gauze safe lamp. (A) shows the three screens of metallic gauze or flame sieves. (B) indicates wires for trimming the wick. Copyright The Royal Society.

Davy's first paper [11], written, read and printed within but weeks of his being alerted to the dangers that coal miners were exposed when working at seams that emitted fire-damp, describes 11 distinct kinds of safety lamps. Two of them are reproduced in figure 1.

Two short paragraphs from Davy's first paper [11], pp. 11–12 also merit citation here:

A candle will burn in a lantern or glass tube made safe with metallic gauze, as well as the open air; I conceive, however, that oil lamps, in which the wick will always stand at the same height, will be preferred.

When the fire-damp is so mixed with the external atmosphere as to render it explosive, the light of the safe lantern or lamp will be extinguished, and warning will be given to the miners to withdraw from, and to ventilate that part of the mine.

**Figure 2.**
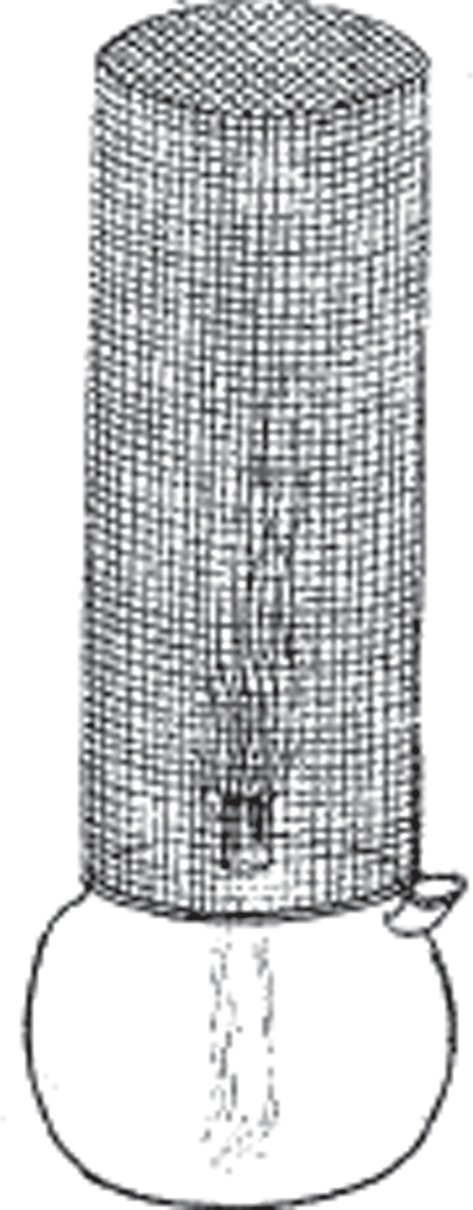
The final form of the safety lamp. Fig. 11 in [11]. Copyright The Royal Society.

Davy's second paper [12], read to the Royal Society on 11 January 1816, explains that wire-gauze could be substituted for the glass sides of the lantern with perfect security, and this realization led him to the final form of the safety lamp (see figure 2).

This additional invention of his consists in covering or surrounding a flame of a lamp or candle by a wire sieve; the coarsest that he tried (with perfect safety) contained 625 apertures in a square inch, and the wire itself was 1/70th of an inch in thickness; and the finest he tried contained 6400 apertures per square inch, with a wire thickness of 1/250th of an inch.

Davy tested the lamp by putting it into explosive mixtures of air and methane (which he called ‘carburetted hydrogen’). When the gas burnt inside the wire-gauze, and even when it became red-hot, explosions never ensued.

## 3. The reaction of the mining communities: further refinements and consequences

In a paper read to the Royal Society on 25 January 1816 [13], Davy announced that two lamps made to his new design (see figure 2) had been tested in the mines of the Wallsend Colliery by Mr John Buddle with complete success. Buddle wrote to Davy in the following terms:

I first tried it in an explosive mixture on the surface, and then took it into a mine…it is impossible for me to express my feelings at the time when I first suspended the lamp in the mine and saw it red hot…I said to those around me: ‘we have at last subdued this monster’.

A few months later, Davy had the satisfaction of seeing his lamp in action in Mr Buddle's pits. Buddle wrote to him on 1 June 1816:

After having introduced your safety lamp into general use in all the collieries under my direction, where inflammable air prevails, and after using them daily in every variety of explosive mixture for upwards of three months, I feel the highest possible gratification in stating to you that they have answered to my entire satisfaction.

Buddle ended his letter with the following words:

It is not necessary that I should enlarge upon the national advantages which must necessarily result from an invention calculated to prolong our supply of mineral coal, because I think them obvious to every reflecting mind; but I cannot conclude without expressing my highest sentiments of admiration for those talents which have developed the properties, and controlled the power, of one of the most dangerous elements which human enterprise has hitherto had to encounter.

Davy was urged, by Buddle and others, to take out a patent to protect his invention which, as Buddle said, would yield him a large income. Davy's reply is highly relevant to those scientists—of which there is now a decreasing number in the academic world—who believe that scientific discovery and invention constitute their own reward. What Davy said was:

My good friend, I never thought of such a thing: my sole object was to serve the cause of humanity; and if I have succeeded, I am amply rewarded in the gratifying reflection of having done so…More wealth could not increase either my fame or my happiness. It might undoubtedly enable me to put four horses to my carriage, but what would it avail me to have it said that Sir Humphry drives his carriage and four?

Davy's lamps were soon in action in many pits; and at a general meeting of coal owners at Newcastle in March 1817, he received a vote of thanks for his great service to the coal miners.

Davy had recognized that if his lamp was exposed to an air current of six to seven feet per second, the flame was blown against the gauze and raised its temperature to the point when it would cause an explosion in dangerous concentrations of fire-damp. This danger he drew to the attention of potential uses; and in later refinements of his safety lamp, there were two shields to protect the flame from a draught. In some lamps, two gauze cylinders to diminish this risk were used. The nature of this risk, and its circumvention, was dealt with at length by Davy in his January 1817 paper [14] and in his monograph [15] published in 1818.

It is noteworthy that Davy's 1817 paper, as alluded to earlier, became the primary foundation stone for the science of combustion and flame [4]. In this paper also, he reported his accidental discovery that a gently heated platinum gauze, foil or wire could bring about the slow combustion of mixtures of vapours (such as ether and alcohol) as well as of coal gas and fire-damp below their ignition temperature. This is one of the first-ever recorded examples of heterogeneous catalysis. It was mentioned by Berzelius in his famous dissertation on catalysis, a word that Berzelius coined [16]. The actual words of Davy's unexpected discovery merit repetition [14, pp. 77–78]:

I was making experiments on the increase of the limits of the combustibility of gaseous mixtures of coal gas and air by increase of temperature. For this purpose, I introduced a small wire-gauze safe-lamp with some fine wire of platinum fixed above the flame into a combustible mixture containing the maximum of coal gas, and when the inflammation had taken place in the wire-gauze cylinder, I threw in more coal gas expecting that the heat acquired by the mixed gas, passing through the wire-gauze would prevent the excess from extinguishing the flame. The flame continued for two or three seconds after the coal gas was introduced; and when it was extinguished, that part of the wire of platinum which had been hottest remained ignited, and continued so for many minutes, and when it was removed into a dark room it was evident that there was no flame in the cylinder.

Davy, later in his article [14, p. 81], states:

I shall now conclude by a practical application. By hanging some coils of fine wire of platinum, or a fine sheet of platinum or palladium above the wick of his lamp, in the wire-gauze cylinder, the coal miner, there is every reason to believe, will be supplied with light mixtures of fire-damp no longer explosive; and should his flame be extinguished by the quantity of fire-damp, the glow of the metal will continue to guide him, and by placing the lamp in different parts of the gallery, the relative brightness of the wire will show the state of the atmosphere in these parts.

Nowadays, it is well known [17] that Pt, Pd (and several other metal) surfaces, when warmed in a Bunsen flame, and then held above the vapour of ether, or alcohol or other volatile organic compounds rich in hydrogen and carbon, will catalyse the oxidation of the vapours and raise the temperature of the metals until they glow.

## 4. A rival inventor

Dr W. R. Clanny, a philanthropic Irish doctor who practised in Sunderland, had seen the tragedies caused by colliery explosions, and in May 1813 he read a paper to the Royal Society on an air-tight lantern containing a candle to which air is supplied from pairs of bellows after bubbling through some water in the bottom of the lantern. The products of combustion escaped through a valve at the top of the lantern for which a water trap was later substituted. Clanny and friends tested his lantern in the most inflammable part of a colliery, where it gave light without causing an explosion. The disadvantage of Clanny's invention was that it needed the services of a boy to carry the lamp and to work the bellows.

The other rival was the redoubtable [18] George Stephenson (1781–1848), the colliery engineer at Killingworth Main, who had noted that the flames of a number of candles placed to the windward of burning blowers of gas were extinguished by the burnt air which was carried towards them. He had also noted that when fire-damp was ignited, it took an appreciable time for the flame to travel from one point to another. This gave him the idea that if a lamp could be made in which the velocity of the mixture of fire-damp and air entering below the flame was sufficient to prevent the explosion pressing downwards, the burnt air would prevent it from pressing upward. Stephenson tested his first lamp successfully in a particularly dangerous part of his colliery on 21 October 1815. His later design, which then became known as ‘the Geordie’, consisted of an oil lamp with a glass chimney. Air was admitted at the side of the lamp through a series of small holes in metal plates, the diameter of the outer hole being 2/25th to 1/22nd of an inch and that of the wires 1/12th to 1/18th of an inch, the burnt gases escaping through a metal cap with small perforations.

Although Stephenson's lamp lacked the precision of detail (on which safety depends) of the Davy lamp, it is remarkable that the keen observations of the unlettered engineer should have led him to a device closely related to Davy's.

In some of the coal mines of the north of England, partly because of local patriotism, the ‘Geordie’ was preferred to the ‘Davy’ lamp. As a consequence of this rivalry, there was some opposition to a proposal (in August 1818) to present Davy with a gift of a plate in token of the miners' and owners' gratitude on the grounds that Stephenson was the first discoverer of the principle of the safety lamp. Davy's supporters carried the day, and when he was in Newcastle in 1817, Sir Humphry was presented with a silver plate at a special banquet. The supporters of Stephenson collected a sum of £1000, which was presented to him with a silver tankard in recognition of the value of the ‘Geordie’.

Subsequently, Davy reacted angrily to Stephenson's claim. He said:

I never heard a word of George Stephenson and his lamp until 6 weeks after my principle of security had been published…he made something like a safe lamp, except that it is not safe, for the apertures below are four times, and those above twenty times too large…there is no analogy between his glass exploding machine and my metallic tissue permeable to light and air and impermeable to flame.

Controversy in newspapers followed. And there was a rumour, reported by Dr Alexander Marcet in a letter to Berzelius, that Davy must have known of Smithson Tennant's unpublished experiments, showing that explosions of mixtures of coal gas and air could not pass through narrow tubes, experiments that Tennant, an English chemist, had made in 1813. The Royal Society stepped in and issued the following statement, signed by Sir Joseph Banks, PRS, W. H. Wollaston, FRS, W. T. Brande, and others:

Sir Humphry Davy not only discovered, independently to all others, and without any knowledge of the unpublished experiments of the late Mr Tennant on flame, the principle of the non-communication of explosions through small apertures, but that he also has the sole merit of having first applied it to the very important purpose of a safety-lamp, which has evidently been imitated in the latest lamps of Mr George Stephenson.

It is gratifying that Michael Faraday, whose honesty and purity of heart are beyond a scintilla of doubt, gave his own abbreviated account of the progress of the invention of the Davy lamp. Faraday's notes for a lecture to the London Philosophical Society in 1817 state:

The great desideratum of a lamp to afford light with safety; several devised; not mention them all but merely refer to that which alone has been found efficacious, the DAVY: this the result of pure experimental deduction. It originated in no accident nor was it forwarded by any, but was the consequence of a regular scientific investigation.

A report of the Select Committee appointed by Parliament in 1835 [17] to enquire into the nature, cause and extent of those lamentable catastrophes that have occurred in the mines of Great Britain praises Davy for his inventiveness and originality. It also exonerates Stephenson of plagiarism.

## 5. Envoi

To express his appreciation of Davy's invention of the safety lamp, Joseph Banks, the President of the Royal Society, had written to Davy declaring that his work would place the Royal Society higher in popular opinion than all other abstruse discoveries (*beyond the understanding of ordinary people*). For his safety lamp, Davy was accorded the Rumford Prize of the Society; and in 1818, he was made a Baronet, the first scientist to be awarded such an honour. (A referee of this paper has drawn to my attention that someone made the comment that if you were responsible for saving the lives of thousands, the reward was a baronetcy, whereas if you were, like Wellington, responsible for the deaths of thousands, the reward was a dukedom.)

**Figure 3.**
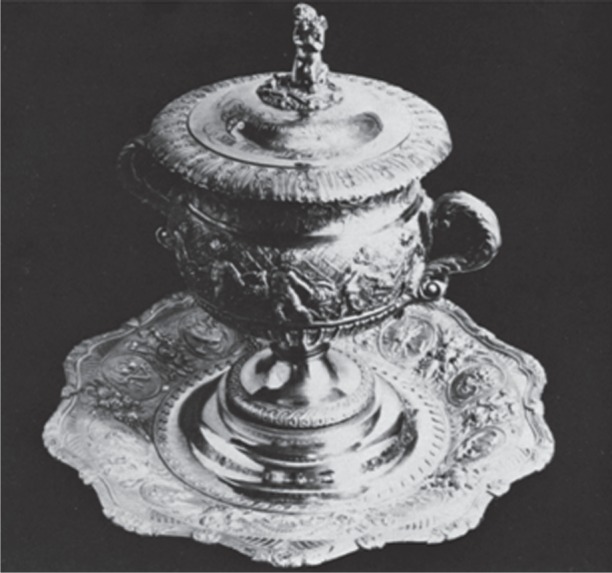
Photo of the silver-gilt salver sent to Humphry Davy by the Tsar of Russia. (From R. King's booklet on Humphry Davy, published by the Royal Institution, 1978.)

Davy's safety lamp was used extensively in the coal mines of Europe, from Flanders to Russia and beyond. Indeed, Tsar Alexander of Russia, in 1825, sent to Davy at the RI a large silver-gilt salver and bowl which was, until recently, used by the director and his wife at dinners given preceding Friday Evening Discourses (figure 3).

Coal miners throughout Britain, up until some 70 years ago, carried their Davy safety lamps with them to and from the collieries. (For countless children who, like myself, belonged to the family of colliers, Davy was the first scientist's name that they encountered. In the coalfields of the north of England, the miners carried the Geordie, not the Davy, lamp.)

Although his health was slowly declining, Davy took up, in the early 1820s, the challenge of arresting the rapid decay of copper sheeting of His Majesty's ships of war, which had prompted the Commissioners of the Navy to consult the Royal Society. On 24 January 1824, he read a paper to the Society entitled:

On the Corrosion of Copper Sheathing by sea-water; and on methods of preventing this effect, and on their application to ships of war and other ships.

On 17 June 1824 (and again on 9 June 1825), Davy read further papers on this topic. Full details of the systematic experiments that Davy, with the assistance of Faraday, carried out in this investigation have been given by Hartley (see [4, pp. 138–139]). Briefly, Davy soldered a piece of tin to 20 times its area of copper and immersed it in seawater to which a little sulfuric acid had been added. The copper remained clean after 3 days, while, in a duplicate experiment, without contact with tin, considerable corrosion had occurred.

Although some considerable practical difficulties were encountered in the application of this discovery of Davy's—known as cathodic protection—it is the method that is now employed worldwide, for ships, submarines and buildings on land, to arrest the corrosion of materials such as iron and steel when exposed to aqueous solutions or gases rich in water vapour.

Davy's other accomplishments are of lasting value: he helped found the Zoological and Geological Societies of London; and when the Athenaeum Club in London was conceived by John Wilson Croker, MP, as *a place of rendezvous* … *for literary men and artists*, Croker first sought support for his proposal from Sir Humphry Davy. Davy chaired the first committee meeting which met at the Royal Society on 16 February 1824, on which day the Club was established (with Michael Faraday as its first secretary).

In 1820, Joseph Banks died, and Davy was elected President of the Royal Society on 30 November that year. At its Anniversary Meeting on 30 November 1826, Davy was re-elected, but it was clear from his speech on that occasion that Davy's health was failing fast. In December of that year, he suffered a stroke. He recovered partially, and in Rome a year later he was again taken seriously ill. His love of natural history, however, never deserted him, and he decided to compose a series of dialogues on the subject entitled *Salmonia*, or *Days of Fly-Fishing*, which became a success. While endeavouring to return to England from his sick bed in Rome, he died in Geneva on 29 May 1829 and was buried there. On his sick bed, he had written *Consolations in Travel, or The Last Days of a Philosopher*, which was published posthumously in 1830.

## Author profile

**John Meurig Thomas** taught and researched for 20 years in the University of Wales (Bangor and Aberystwyth) before taking up the Headship of the Department of Physical Chemistry, University of Cambridge, and a Professorial Fellowship at King's College there in 1978. In 1986, he was appointed Director of the Royal Institution of Great Britain, and of the Davy Faraday Research Laboratory, London. In 1993, he became Master of Peterhouse, University of Cambridge, and Honorary Professor of Materials Science, Cambridge. His early work as a solid-state and surface chemist was concerned with the chemical consequences of dislocations and other structural faults in various crystals—layered minerals, inorganic hydrates and organic molecular solids—and with the development of electron microscopy as an indispensable chemical technique. From the 1980s onwards, he has designed, characterized (*in situ*) and discovered numerous new solid catalysts. His research has earned him many awards: the Willard Gibbs, Pauling and Zewail Gold Medals from the USA, the Kapitza and Semenov medals from the Russian Academy, the Natta Gold Medal from the Italian Chemical Society, the Davy Medal from the Royal Society, the Faraday Medal (and six others) from the Royal Society of Chemistry and the Blaise Pascal Medal for Materials Science, 2014, from the European Academy of Sciences.

For his contributions to geochemistry, a new mineral, meurigite, was named in his honour. He was knighted for services to chemistry and the popularization of science.

His interest in Davy and his lamp was aroused at an early age: his brother (12 years his senior) worked underground for 50 years and his father for 30 years in the coal mines of southwest Wales.
